# γ-Glutamyl transferase 7 is a novel regulator of glioblastoma growth

**DOI:** 10.1186/s12885-015-1232-y

**Published:** 2015-04-07

**Authors:** Timothy T Bui, Ryan T Nitta, Suzana A Kahn, Seyed-Mostafa Razavi, Maya Agarwal, Parvir Aujla, Sharareh Gholamin, Lawrence Recht, Gordon Li

**Affiliations:** 1 Department of Neurosurgery, Stanford University School of Medicine, Institute for Stem Cell Biology and Regenerative Medicine, 1201 Welch Rd P309, Stanford, CA 94305-5487 USA; 2 Institute for Stem Cell Biology and Regenerative Medicine, Stanford University School of Medicine, Stanford, CA USA; 3 Department of Neurology and Neurological Sciences, Stanford University School of Medicine, Stanford, CA USA

**Keywords:** γ-Glutamyl transferase, γ-Glutamyl transferase 7, Reactive oxygen species, Glioblastoma

## Abstract

**Background:**

Glioblastoma (GBM) is the most malignant primary brain tumor in adults, with a median survival time of one and a half years. Traditional treatments, including radiation, chemotherapy, and surgery, are not curative, making it imperative to find more effective treatments for this lethal disease. γ-Glutamyl transferase (GGT) is a family of enzymes that was shown to control crucial redox-sensitive functions and to regulate the balance between proliferation and apoptosis. GGT7 is a novel GGT family member that is highly expressed in brain and was previously shown to have decreased expression in gliomas. Since other members of the GGT family were found to be altered in a variety of cancers, we hypothesized that GGT7 could regulate GBM growth and formation.

**Methods:**

To determine if GGT7 is involved in GBM tumorigenesis, we modulated GGT7 expression in two GBM cell lines (U87-MG and U138) and monitored changes in tumorigenicity *in vitro* and *in vivo*.

**Results:**

We demonstrated for the first time that GBM patients with low GGT7 expression had a worse prognosis and that 87% (7/8) of primary GBM tissue samples showed a 2-fold decrease in GGT7 expression compared to normal brain samples. Exogenous expression of GGT7 resulted in a 2- to 3-fold reduction in proliferation and anchorage-independent growth under minimal growth conditions (1% serum). Decreasing GGT7 expression using either short interfering RNA or short hairpin RNA consistently increased proliferation 1.5- to 2-fold. In addition, intracranial injections of U87-MG cells with reduced GGT7 expression increased tumor growth in mice approximately 2-fold, and decreased mouse survival. To elucidate the mechanism by which GGT7 regulates GBM growth, we analyzed reactive oxygen species (ROS) levels in GBM cells with modulated GGT7 expression. We found that enhanced GGT7 expression reduced ROS levels by 11-33%.

**Conclusion:**

Our study demonstrates that GGT7 is a novel player in GBM growth and that GGT7 can play a critical role in tumorigenesis by regulating anti-oxidative damage. Loss of GGT7 may increase the cellular ROS levels, inducing GBM occurrence and growth. Our findings suggest that GGT7 can be a promising biomarker and a potential therapeutic target for GBM.

**Electronic supplementary material:**

The online version of this article (doi:10.1186/s12885-015-1232-y) contains supplementary material, which is available to authorized users.

## Background

Glioblastoma (GBM) is the most common and aggressive malignant primary brain tumor in humans, comprising 60-75% of all astrocytomas and 17% of all primary intracranial tumors [1]. Despite multimodal treatment options, the prognosis for GBM patients is extremely poor, with a median survival time of ~16 months [2-4]. Because of the severity and lethality of this disease, identifying novel pathways is paramount in developing more effective therapies.

The GGT family is comprised of 13 enzymes that are involved in glutathione metabolism and whose expressions are altered in numerous human malignancies including breast, uterine, and lung cancer and leukemia [5,6]. The GGT family modulates crucial redox sensitive functions such as anti-oxidant/anti-toxic defense and cellular proliferative/apoptotic balance suggesting that it plays an important role in tumor progression, invasion, and drug resistance [7]. Currently, there are no reports linking GGT to GBM.

GGT7 (formerly GGTL3, GGTL5) is a novel member of the GGT family that has not been widely studied. GGT7 shares a 47% and 52% similar amino acid sequence to its more well characterized family members GGT1 and GGT5, respectively [6]. While similar, GGT7 may have a novel function as a result of the high variation in its light chain, compared with GGT1 and GGT5, resulting in altered substrate binding. This discrepancy was evident when only GGT7 was found to interact with proteins associated with lung cancer, indicating it could play a role in cancer progression [8]. GGT7 also differs from the other GGT isoforms, since it is the only isoform to have ~20-fold higher mRNA expression in the brain compared with other normal tissues [9] and has decreased expression in gliomas compared with the normal brain [10]. These previous findings suggest that GGT7 could play an important role in GBM growth.

To determine if GGT7 is involved in GBM tumorigenesis, we modulated GGT7 expression in two GBM cell lines, U87-MG and U138. We determined that transduction of GBM cells with GGT7 resulted in a 2-3-fold reduction in both proliferation and anchorage-independent growth when the cells were cultured under low serum (1%) conditions, demonstrating that GGT7 regulates GBM growth. To confirm these findings, GGT7 expression was reduced by introducing short interfering RNA (siRNA) or short hairpin RNA (shRNA) specific to *GGT7*. Reducing GGT7 expression using siRNA or shRNA increased GBM cell proliferation *in vitro*. Consistently, intracranial injections of U87-MG cells stably transduced with shRNA to GGT7 resulted in an increase in tumor size and a decrease in mouse survival. To elucidate the mechanism by which GGT7 regulates GBM tumorigenesis, we analyzed the ability of GGT7 to modulate crucial redox-sensitive cellular functions. We demonstrated that enhanced expression of GGT7 reduced the levels of reactive oxygen species (ROS) by 11-33%, indicating that GGT7 can protect cells from oxidative damage. Our study demonstrates for the first time that GGT7 plays an important role in GBM proliferation and could be a novel biomarker and therapeutic target for GBM.

## Methods

### Cell lines and primary GBM tissue

Cell lines U87-MG and U138 (American Type Culture Collection, Manassas, VA) were cultured in Dulbecco’s modified Eagle medium (DMEM), supplemented with 10% or 1% fetal bovine serum (FBS) and 2 mM L-glutamine. All cells were maintained at 37°C in 5% CO_2._ Primary GBM and normal brain samples were provided by Samuel Cheshier, MD. The normal brain was harvested from the temporal lobe of a patient undergoing anterior temporal lobectomy for removal of mesial temporal sclerosis. The tissue was immediately frozen in dry ice and later thawed for harvesting of protein lysate. Human specimens were obtained from adult patients who signed an informed consent. The study protocol was approved by Stanford University Human Subjects Research and Institutional Review Board (IRB-18672). Phoenix cells were kindly provided by the Nolan lab (Stanford University) and cultured under similar conditions as stated above.

### Kaplan-Meier survival curve

The Kaplan-Meier survival curve was derived using the National Cancer Institute REMBRANDT data source. The GBM patients were separated into high or low GGT7 expression and graphed according to patient survival. The survival curves were derived using the http://genedesk.ucsd.edu/home/ website. Log-rank test was used to determine the statistical significance.

### Gene expression profiling from primary GBM samples

For two GBM tumor panels and one normal brain panel, we used the Affymetrix U133 Plus 2.0 Microarray (Affymetrix, Santa Clara, CA) to analyze mRNA expression in primary samples. An analysis by Murat *et al*. [11] contained 84 GBM samples derived from primary tumors and an analysis by Lee *et al.* [12] contained 101 primary GBM samples. The normal brain gene expression profile contained 173 samples for different regions of the brain, including the hippocampus, entorhinal cortex, superior frontal gyrus, and postcentral gyrus [13]. The expression data were normalized with the MAS5.0 algorithm within the Affymetrix GCOS program. All data were analyzed using the R2 bioinformatic tool (http://r2.amc.nl). The expression was transformed to 2log and graphed as a boxplot. The single factor analysis of variance was used to compare the means of the different groups and determine the statistical significance.

### Western blot

For protein analysis, protein extracts from cells were harvested and immunoblotted as previously described [14]. The following antibodies were used for immunoblotting: GGT7 (ab129395; Abcam, Cambridge, MA) and glyceraldehyde 3-phosphate dehydrogenase (GAPDH) (14C10; Cell Signaling Technology, Beverly, MA). Enhanced Chemiluminescence Substrate (PerkinElmer, Waltham, MA) and Gene GNOME (Syngene, Frederick, MD) were used for visualization. Chemiluminescence signals were quantitated using NIH Image J (National Institutes of Health, Bethesda, MD). All experiments were conducted in triplicate where a representative image was used to demonstrate the findings. The statistical significance was determined using 2-sided *t* tests.

### Retroviral and lentiviral infections

The retrovirus pMSCV-YPet was generated by subcloning YPet from the pCEP4-YPet plasmid into the pMSCV backbone. Retroviral infections were carried out as previously described [14]. Thirty-six hours after infection, the infected cells were selected by culturing for 2 days in selective medium containing 0.5 μg/mL puromycin. Lentiviral infections were conducted using the pTRIPZ shGGT7 plasmid (RHS4696-200683561; Thermo Fisher Scientific, Waltham, MA) in a manner similar to that described with the retrovirus, except the packaging plasmids, psPAX2 and pMD2.G, and Mirus TransIT-LT1 (MIR2300; Mirus Bio?, Madison, WI) were used. Thirty-six hours after infection, the cells were selected using 0.5 μg/ml puromycin for 3 days.

### RNA interference

U87-MG and U138 cells were transfected with 25 nM GGT7 (SI00427126; Qiagen, Valencia, CA) or non-specific control siRNA (4390843; Ambion Inc., Austin TX) for 24 h, using DharmaFECT transfection reagent 1 (T-2001-02; Thermo Fisher Scientific), according to the manufacturer’s protocol. The lentiviral inducible shRNA plasmid, pTRIPZ, was used to express shRNA to the gene GGT7.

### Cell growth analysis

U87-MG- and U138-infected cells were plated in six-well plates (5 × 10^5^ cells per well) and cultured in DMEM supplemented with 10% or 1% FBS. The number of live cells was counted daily for several days using the trypan blue exclusion assay or cell titer blue assay. On the last day, collected cells were subsequently harvested and subjected to Western blot analysis to determine protein expression. Experiments were done in triplicate and results are expressed as mean ± SD. The statistical significance was determined using a 2-sided *t* test.

### Soft agar assay

U87-MG-infected cells were plated in six-well plates (3 × 10^5^ cells per well) and suspended in DMEM with 10% or 1% FBS as previously described [14]. The presence of colonies was scored after 10 days using Genetools software (Syngene) or counted manually with a compound light microscope. Experiments were done in triplicate and results are expressed as mean ± SD. The statistical significance was determined using a 2-sided *t* test.

### Detecting cellular ROS

U87-MG- and U138-infected cells were plated in a 96 well plate (3 × 10^3^ cells per well) and were suspended in DMEM with 10% or 1% FBS. After 24 h, cells were stained with DCFDA according to the manufacturer’s protocol (ab113851; Abcam). TBHP was used to induce ROS damage. ROS levels were normalized per cell count using cell titer blue to stain cells for 3 h. Fluorescence was read with a FLUOstar Omega plate reader at Ex 485 nm/Em 520 nm for DCFDA, and at Ex 544 nm/Em 590 nm for cell titer blue. Experiments were done in triplicate and results are expressed as fold change ± SD. The statistical significance was determined using a 2-sided *t* test.

### Generation of intracranial xenografts

100,000 U87-MG shGGT7 cells were intracranially transplanted into 6-8-week-old NSG mouse brains using a stereotactic frame 2-mm posterior to the bregma, 2-mm lateral to the midline, and 3–4 mm deep with respect to the skull. Mice were monitored daily until overt neurological defects were observed. The brains were then harvested for analysis. 10 mice per treatment group and the statistical significance were determined using a log rank test.

### Bioluminescent imaging

Bioluminescent imaging was performed 15 days after U87 shGGT7 cells were injected on an IVIS Spectrum (Caliper Life Science) and quantified using Living Image 4.0 software. D-Luciferin (firefly) potassium salt solution (Biosynth, Itasca, IL) was prepared (16 mg/mL) and injected intraperitoneally (0.139 g luciferin per kilogram body weight). Total luminescence (photons per second) was obtained by imaging mice until peak radiance was achieved.

## Results

### GBM patients with decreased GGT7 expression have worse prognoses

**Figure 1:**
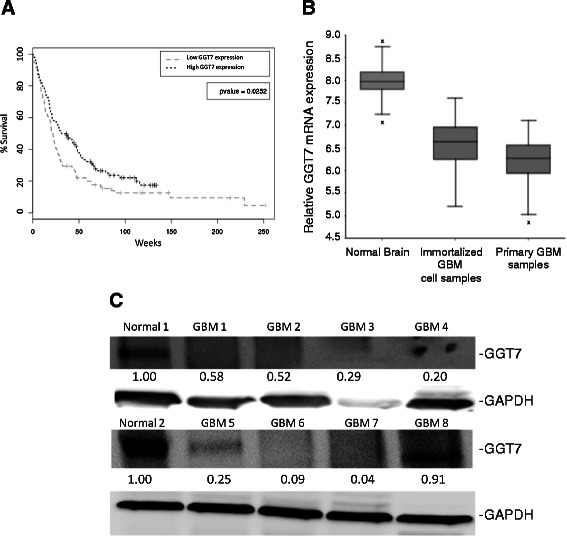
**Survival data and expression levels of GGT7 in GBM patients. (A)** Patients were separated by relative expression of GGT7. Statistical significance was determined using log-rank test. **(B)** Expression of GGT7 using the R2 microarray analysis and visualization platform (http://r2.amc.nl). Normal brain samples consisted of 173 tissue samples, GBM cell samples consisted of 84 cell lines derived from GBM patients, and the primary GBM samples consisted of 101 primary GBM tissue samples. *-denotes a sample that resided outside the 95% confidence interval. **(C)** Western blot analyzing GGT7 expression in eight primary GBM tissue samples and two normal brain samples. GAPDH was used as a protein load control. Fold difference is represented below each blot.

To determine if GGT7 is involved in GBM proliferation, we conducted a preliminary prognosis analysis of GGT7 expression in GBM patients using the Repository of Molecular Brain Neoplasia Data (REMBRANDT). After sorting the GBM patients into high or low GGT7 expression, our findings suggest that GBM patients with high GGT7 expression had a better prognosis compared with their low-expressing counterparts (*P* = 0.02) (Figure 1A). This initial finding suggests that loss of GGT7 may contribute to the growth of GBM.

### GBM primary samples have reduced expression of GGT7

Since GGT7 is the only GGT family member highly expressed in the brain to any extent, we compared its expression between primary GBM tissue and normal brain [9]. The analysis was done using the R2 microarray analysis and visualization platform (http://r2.amc.nl). The platform analyzed previously conducted microarray expression data and normalized expression levels with the MAS5.0 algorithm within the Affymetrix GCOS program. The expression was then transformed to 2log and graphed as a boxplot. The single factor analysis of variance was used to compare the means of the different groups and determine the statistical significance. We discovered that, compared with an expression data set containing 172 normal brain sections, GGT7 expressionwas significantly increased when compared with a dataset derived from 84 primary GBM patient samples (*P* = 3.7 × 10^-10^) (Figure 1B). We also analyzed a data set that contained 101 GBM cell samples that were derived from GBM patients [12]. We consistently saw a reduction in GGT7 expression that was statistically significant (*P* = 1.3 × 10^−8^) when compared with normal brain. To determine if the decrease was specific to GGT7, we also analyzed GGT1 and GGT5 expression. We discovered that GGT1 had a modest reduction in the immortalized GBM cell data set, but no significant change in the primary GBM samples (Additional file 1: Figure S1). GGT5 had no significant change in either data set compared with the normal brain samples (Additional file 1: Figure S1). The microarray findings were confirmed by analyzing GGT7 protein expression in eight primary GBM samples. We demonstrated that 87.5% (7/8) of the GBM samples had a ~2- to ~25-fold decrease in GGT7 protein expression compared with two normal brain samples (Figure 1C). The two normal brain samples had a similar GGT7 expression with only a 1.08-fold difference (Data not shown). Our findings suggest that loss of GGT7 correlates with enhanced GBM growth.

### Overexpression of GGT7 decreased GBM tumorigenic phenotypes

**Figure 2:**
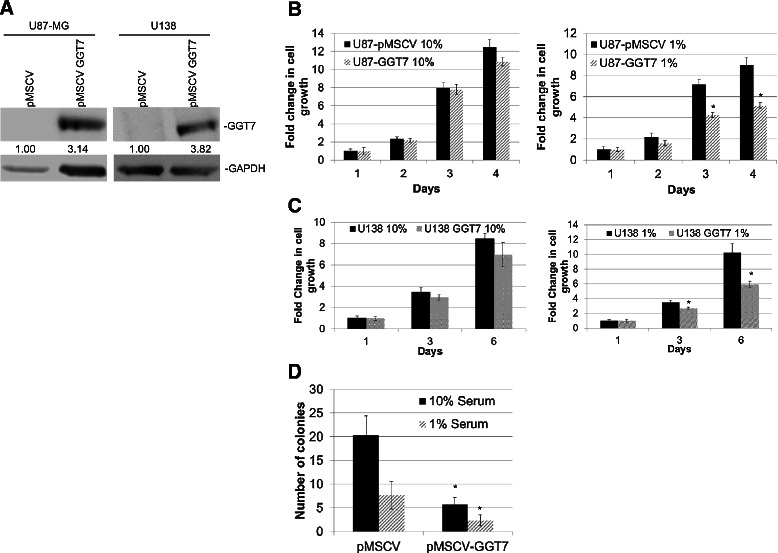
**Reducing GGT7 expression increases GBM cell growth*in vitro*. (A)** siRNA to GGT7 (siGGT7) reduced endogenous GGT7 protein expression compared with the siScram. GAPDH was used as a protein load control. Fold difference is represented below each blot. **(B)** Growth of U138 cells with reduced GGT7 expression under normal (10% serum) conditions. **(C)** Cell growth under low nutrient (1% serum) conditions. **(D)** Anchorage-independent growth assays were performed under normal and low nutrient conditions. *Denotes a statistically significant change, *P* < 0.05, measured by the Mann–Whitney U test.

**Figure 3:**
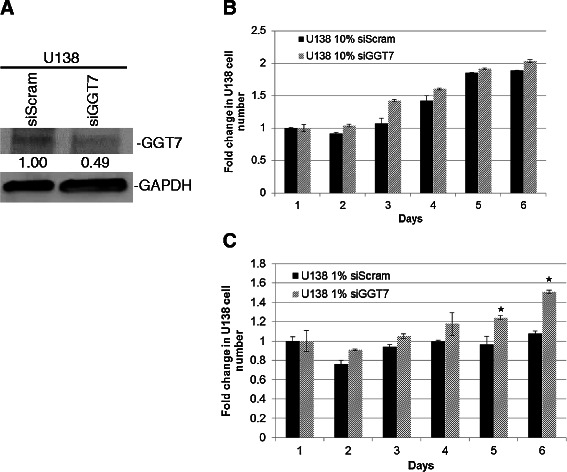
**GBM cells transduced with GGT7 have decreased cell growth. (A)** Western blot of GBM cells stably expressing exogenous GGT7. GAPDH was used as a protein load control. Fold difference is represented below each blot. **(B)** Cell proliferation of U87 cells transduced with GGT7 cultured under normal (10% serum, *left*) and low nutrient (1% serum, *right*) conditions. **(C)** Proliferation of U138-GGT7 cultured under normal (10% serum, *left*) and low nutrient (1% serum, *right*) conditions. *Denotes a statistically significant change, *P* < 0.05, measured by the Mann–Whitney U test.

To determine if GGT7 plays an important role in GBM tumorigenesis, we generated two immortalized GBM cell lines (U87-MG and U138) that were stably transduced with GGT7. Western blot analysis confirmed that U87-MG cells expressed exogenous GGT7 (U87-pMSCV GGT7) compared with the control vector-infected cells (U87-pMSCV) (Figure 2A). We also observed a 3-fold increase in GGT7 expression in the U138 infected cells (U138-GGT7) compared with the control cells (U138-pMSCV) (Figure 3A). Enhanced expression of GGT7 had no significant effect on the proliferation of U87-GGT7 cells compared with the control cell line when the cells were cultured under normal serum conditions (10% serum) (Figure 3B). When the cells were cultured with 1% serum, however, we observed a 2-fold decrease in cell growth, suggesting that GGT7 can regulate GBM growth when the cells are metabolically stressed. We observed a similar trend when the U138-GGT7 cells were cultured under normal serum conditions and serum-starved conditions (Figure 3C). To expand on these initial findings, we also studied the ability of U87-GGT7 cells to grow under anchorage-independent conditions. We discovered that these cells grown with 1% serum had a 3-fold decrease in the number of colonies compared with the control cells (Figure 3D). A 3-fold reduction in the number of colonies was also observed when the U87-GGT7 cells were grown under normal conditions (Figure 3D). Our data suggest that when GBM cells are metabolically stressed, enhanced expression of GGT7 decreases GBM tumorigenesis.

### Reduced GGT7 expression increased GBM cell proliferation

**Figure 4:**
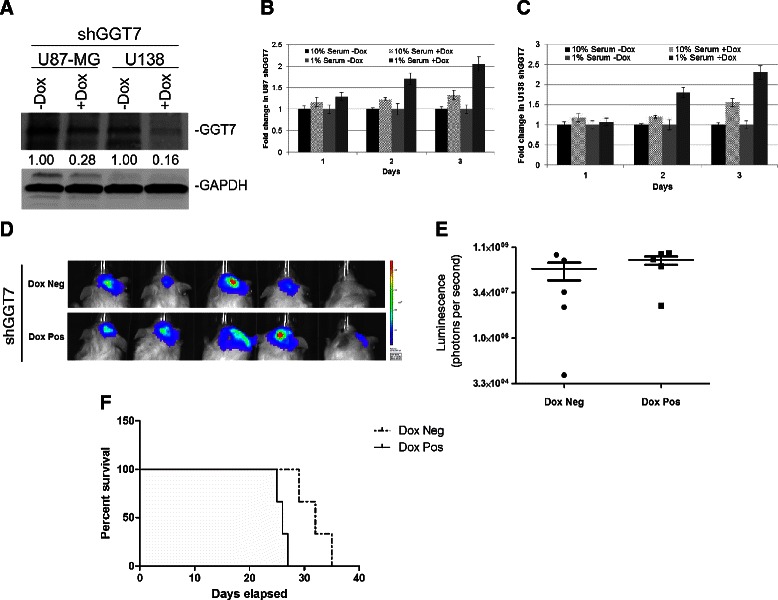
**Reducing GGT7 expression increases GBM cell growth*in vivo*. (A)** GBM cells with a Dox-inducible shRNA to GGT7 (shGGT7) reduced endogenous GGT7. GAPDH was used as a protein load control. Fold difference is represented below each blot. Cell growth of U87 **(B)** and U138 **(C)** shGGT7 cells induced with Dox under normal nutrient (10% serum) and low nutrient (1% serum) growth conditions. **(D)** NSG mice were injected with U87 shGGT7 cells transduced with green fluorescent protein-luciferase. Luminescent mouse images were obtained 15 days after injection. **(E)** Luminescence levels were shown using a box-whisker plot showing quantitated luminescence (photons/sec) levels. **(F)** Survival curve of mice injected with U87 shGGT7 cells treated with Dox (Dox pos) or vehicle control (Dox neg).

To confirm the importance of GGT7 in GBM growth, we reduced GGT7 expression using siRNA specific to *GGT7* (siGGT7). Introduction of siGGT7 into U138 reduced expression of endogenous GGT7 by 2-fold compared with the scramble control (siScram) resulting in a 1.5-fold increase in cell growth under low serum conditions (1%), and a minimal change when the cells were grown under 10% serum conditions (Figure 2A-C). We verified this finding by reducing exogenous GGT7 expression in the U138-GGT7 cells. We demonstrated that reducing exogenous GGT7 expression 5-fold restored cell proliferation to a similar level as the uninfected U138 cells (Additional file 2: Figure S2A and B). Since siRNAs can have off-target effects, we generated a shRNA inducible to GGT7 in U87-MG and U138 cells (shGGT7) to confirm our siGGT7 finings. Upon doxycycline (Dox) induction (shGGT7 + Dox), GGT7 protein expression was reduced ~3- and ~6-fold in U87-MG and U138 shGGT7 cells, respectively, compared with the uninduced control (shGGT7 -Dox) cells (Figure 4A). The U87 shGGT7 + Dox cells had a small increase in growth under normal growth conditions (10% serum) (1.4-fold), while a more significant increase was observed under 1% serum conditions (2.1-fold) (Figure 4B). We observed similar findings in U138 shGGT7 cells. Proliferation of these cells was increased by 1.5-fold when the cells were cultured with 10% serum and 2.5-fold with 1% serum (Figure 4C).

### Decreased expression of GGT7 increased tumor growth and reduced mouse survival

To determine if GGT7 plays a functional role in GBM tumorigenesis, we conducted *in vivo* tumor growth assays in NOD SCIDγ (NSG) mice. To monitor GBM growth *in vivo,* we stably transduced U87 shGGT7 cells with green fluorescent protein-luciferase. We pretreated the U87 shGGT7 cells with Dox and performed intracranial injections into the NSG mice. After injection of the cells the mice were not given additional Dox. The mice were injected with 100,000 U87 shGGT7 + Dox or U87 shGGT7 -Dox cells. Tumor growth after implantation was monitored using the IVIS (Caliper Life Sciences, Alameda, CA) *in vivo* live imaging system as measured by luminescence levels. Fifteen days after injection, mice with U87 shGGT7 + Dox cells presented a 2-fold increase in luminescence compared with the shGGT7 -Dox controls. Although the findings were not statistically significant (P = 0.08), our data strongly suggest that decreased expression of GGT7 increases GBM tumor growth *in vivo* (Figure 4D,E). To expand on this finding, we also monitored post-injection survival. The mice injected with U87 shGGT7 + Dox cells had a lower survival compared with the mice injected with U87 shGGT7 -Dox cells, with the median survival time after tumor implantation increasing from 25 days to 33 days (*P* = 0.02) (Figure 4F).

### GGT7 protected cells from ROS activity

**Figure 5:**
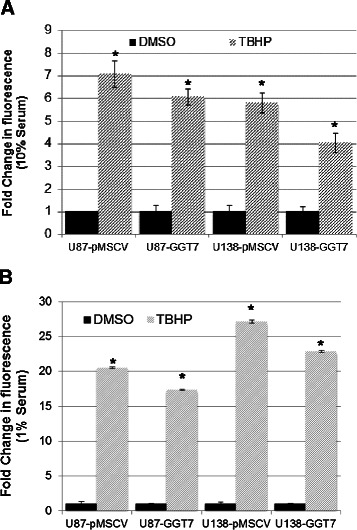
**ROS are decreased with increased levels of GGT7.** GBM cells transduced with GGT7 were labeled with DCFDA (20 μM) or unlabeled **(**phosphate-buffered saline**)** and then cultured with 50 μM TBHP and Dimethyl sulfoxide (DMSO) as a control. Cells were then analyzed on a fluorescent plate reader. **(A)** Cells cultured under normal growth conditions (10% serum). **(B)** Cells grown under low nutrient (1% serum) conditions. * Denotes a statistically significant change, *P* < 0.05, as measured by the Mann–Whitney U test.

Previous research has shown that the GGT family can regulate the cell’s ability to handle oxidative stress [15]. To determine whether GGT7 functions as an anti-oxidant, we monitored ROS activity using a fluorescent assay. The GBM cells transduced with GGT7 were treated with tert-butyl hydroperoxidase (TBHP), a known ROS inducer, and ROS levels were measured using 2’,7’-dichlorofluorescin diacetate (DCFDA). We discovered that under normal growth conditions (10% serum), U87-GGT7 cells had a 15% reduction in ROS activity compared with the control cells treated with TBHP, while U138-GGT7 cells demonstrated a 33% reduction when compared with the control cells (Figure 5A). When we analyzed our cells under 1% serum conditions we discovered that ROS levels were ~3-fold higher than the levels under 10% serum conditions in all the cell lines. This finding was consistent with previous reports demonstrating that serum deprivation conditions increase ROS activity [16]. Surprisingly we observed a similar decrease in ROS activity under low serum conditions, culminating in a 15% reduction in U87-GGT7 cells and a 20% decrease in U138-GGT7 cells (Figure 5B). Our findings indicate that GGT7 could play an important role in the anti-oxidative defense of cells.

## Discussion

Little is known about the function of GGT7 or whether it plays a role in tumorigenesis. We demonstrated for the first time that GBM patients with low GGT7 expression had a worse prognosis and that 87% of the primary GBM samples tested had reduced GGT7 expression (Figure 1A). By modulating GGT7, we demonstrated that the loss of this enzyme increased cellular proliferation and *in vivo* tumor growth and decreased survival in mice injected intracranially with U87-MG cells (Figure 4). Cells transduced with GGT7 consistently had decreased levels of growth (Figure 3). These growth changes were more evident when the cells were cultured under low serum conditions (Figure 3). To elucidate the mechanism by which GGT7 could be regulating GBM tumorigenesis, we studied ROS levels in our cells with modulated GGT7 expression. We found that enhanced GGT7 expression reduced ROS levels, indicating that GGT7 might behave as a regulator of oxidative damage (Figure 5). Our study demonstrates for the first time that GGT7 is a novel player in GBM growth and that GGT7 plays a critical role in tumorigenesis by regulating the anti-oxidative damage occurring within the tumor cells.

There is growing awareness that oxidative stress can play a major role in cancer [17]. ROS can cause oxidative damage to tumor suppressor genes and enhance expression of proto-oncogenes. Moreover, oxidative stress has been shown to induce malignant transformation of cells in culture [18,19]. ROS are potential carcinogens because they facilitate mutagenesis, tumor promotion and progression. In addition, increased levels of ROS were found to have growth-promoting effects through redox-responsive cell signaling cascades. Normal and tumor cells were shown to have increased proliferation and expression of growth-related genes if exposed to elevated ROS levels [20]. Our findings suggest that GGT7 could play an integral role in GBM tumorigenesis by regulating the oxidative damage that occurs within glial cells. Loss of GGT7 could increase the levels of ROS damage, thereby increasing the occurrence or growth of GBM, resulting in the worse prognosis we observed (Figure 5).

The role of GGT in oxidative stress is well studied and provides some interesting questions. Previous studies suggest that the GGT family could exert both pro- and anti-oxidant effects [15]. GGT was shown to play a key role in glutathione homeostasis by breaking down extracellular glutathione and providing cysteine, the rate-limiting substrate for intracellular synthesis of glutathione [21]. Glutathione is a well-established anti-oxidant, and levels of intracellular glutathione increased by GGT were found to decrease ROS levels [22]. However, GGT was shown to also produce cysteinyl-glycine through cleavage of extracellular glutathione. Cysteinyl-glycine can reduce ferric iron (Fe^3+^) to ferrous iron (Fe^2+^), resulting in an iron redox cycle that produces ROS [23]. The contradictory functions of GGT may be attributable to each GGT family member having a different enzymatic function. Structurally, GGT is composed of a heavy chain, which anchors the enzyme to the cell membrane, and a light chain, which regulates GGT enzymatic activity through glutathione binding. The difference between GGT7 and GGT1/5 occurs in the light chain, indicating that GGT7 might be able to bind extracellular glutathione with greater affinity, allowing for more reuptake of glutathione, thereby generating more intracellular glutathione and thus, greater antioxidant activity [6].

In addition, GGT7 may play a role in regulating GBM tumorigenesis beyond monitoring cellular thiol metabolism. Previous research has shown that modulating GGT1/5 expression will alter growth capacity, but these effects were not attributed to changes in cysteine or glutathione levels [24]. Instead GGT7 may protect GBM cells by inactivating other harmful metabolites that accumulate during cellular growth or by regulating as of yet unknown oncogenic protein. Additional studies are needed to determine if GGT7 can play a protective role outside of regulating cellular ROS levels.

## Conclusions

Through our study, we demonstrate that GGT7 can play a key role in regulating GBM growth and survival *in vitro* and *in vivo*. In addition, we have elucidated the mechanism by which GGT7 can prevent tumorigenesis by regulating ROS levels within GBM cells. Loss of GGT7 would lead to increased ROS activity, and in turn, stimulate tumorigenic phenotypes. Our findings are novel because they show for the first time that GGT7 can be used as a biomarker for GBM prognosis, and as a potential therapeutic target by reducing oxidative damage within cells.

## Additional files

Additional file 1: Figure S1. Expression of GGT1 and GGT5 in primary GBM samples. Expression of GGT1 (A) and GGT5 (B) using the R2 microarray analysis and visualization platform (http://r2.amc.nl). Normal brain samples consisted of 173 tissue samples, GBM cells samples consisted of 84 cell lines derived from GBM patients, and the primary GBM samples consisted of 101 primary GBM tissue samples. *-denotes a sample that resided outside the 95% confidence interval.
Additional file 2: Figure S2. Reducing exogenous GGT7 expression increases GBM cell growth *in vitro*. (A) siRNA to GGT7 (siGGT7) reduced exogenous GGT7 expression compared with scrambled control (siScr). GAPDH was used as a protein load control. Fold difference is represented below each blot. (B) Cell growth of U138-GGT7 with reduced expression of GGT7 under low nutrient growth conditions.

## Abbreviations

GBM: Glioblastoma
GGT: γ-glutamyl transferase
siRNA: short interfering RNA
shRNA: short hairpin RNA
ROS: Reactive oxygen species
siGGT7: siRNA specific to *GGT7*
siScram: scramble control
shGGT7: shRNA inducible to GGT7
Dox: Doxycycline
NSG: NOD SCIDγ
TBHP: Tert-butyl hydrogen peroxide
DCFDA: 2’,7’-dichlorofluorescein diacetate
TNFα: Tumor necrosis factor α
IL-12: Interleukin-12
DMEM: Dulbecco’s modified Eagle medium
FBS: Fetal bovine serum
GAPDH: Glyceraldehyde 3-phosphate dehydrogenase
