# Supplementary material for: γ-Glutamyl transferase 7 is a novel regulator of glioblastoma growth
Source: BMC Cancer. 2015 Apr 7;15:225. doi: 10.1186/s12885-015-1232-y (PMC4393868; doi:10.1186/s12885-015-1232-y)
Supplement: Additional file 1: Figure S1. — Expression of GGT1 and GGT5 in primary GBM samples. Expression of GGT1 (A) and GGT5 (B) using the R2 microarray analysis and visualization platform (http://r2.amc.nl). Normal brain samples consisted of 173 tissue samples, GBM cells samples consisted of 84 cell lines derived from GBM patients, and the primary GBM samples consisted of 101 primary GBM tissue samples. *-denotes a sample that resided outside the 95% confidence interval. [file 12885_2015_1232_MOESM1_ESM.pptx]

## Slide 1
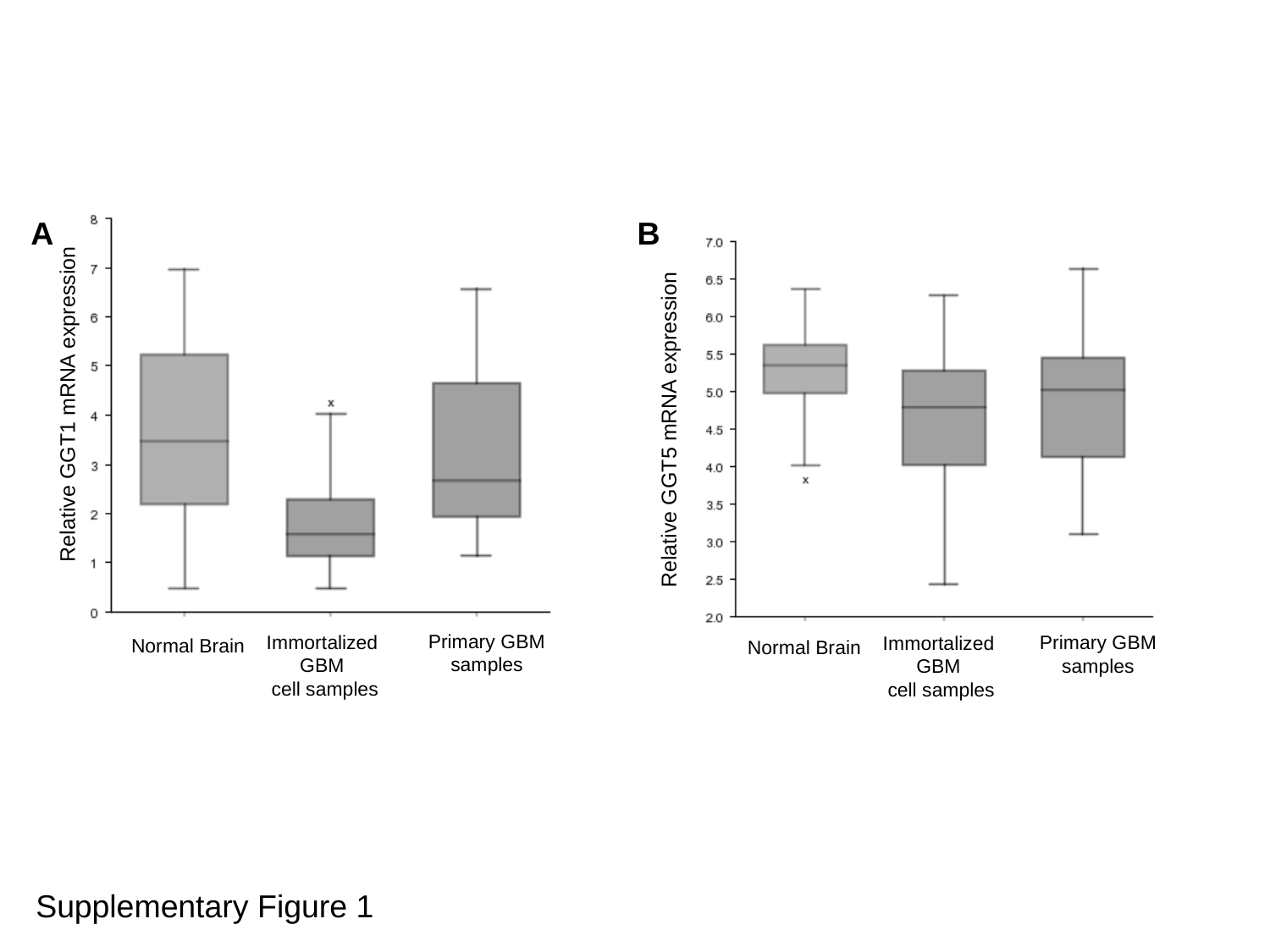

A
B
Relative GGT1 mRNA expression
Relative GGT5 mRNA expression
Primary GBM
samples
Immortalized
GBM
cell samples
Primary GBM
samples
Immortalized
GBM
cell samples
Normal Brain
Normal Brain
Supplementary Figure 1
