# Supplementary figures and images for: γ-Glutamyl transferase 7 is a novel regulator of glioblastoma growth
Source: BMC Cancer. 2015 Apr 7;15:225. doi: 10.1186/s12885-015-1232-y (PMC4393868; doi:10.1186/s12885-015-1232-y)

## Slide 1
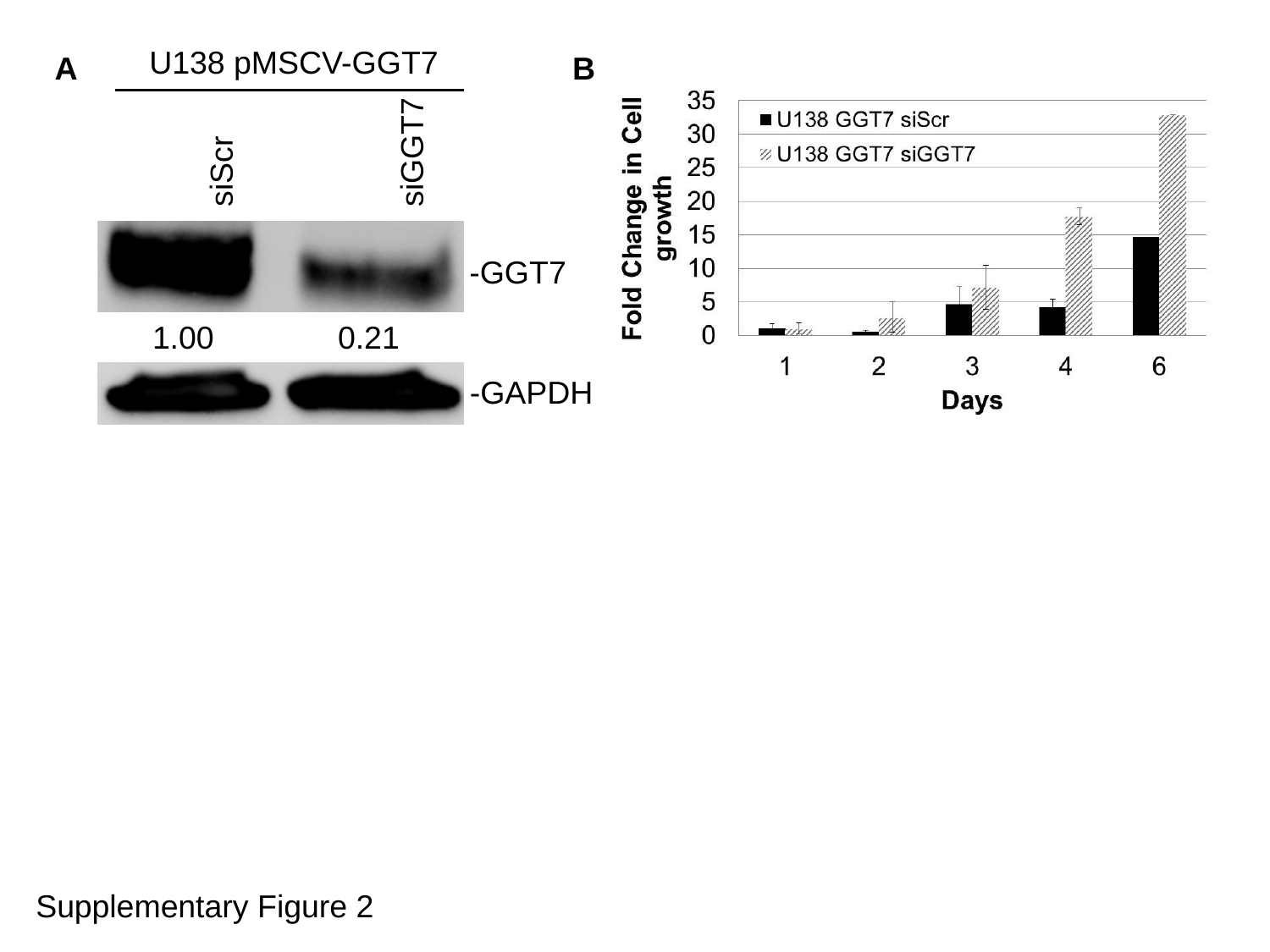

siScr
siGGT7
U138 pMSCV-GGT7
-GGT7
1.00 0.21
-GAPDH
A
B
Supplementary Figure 2

Supplement: Additional file 2: Figure S2. — Reducing exogenous GGT7 expression increases GBM cell growth in vitro. (A) siRNA to GGT7 (siGGT7) reduced exogenous GGT7 expression compared with scrambled control (siScr). GAPDH was used as a protein load control. Fold difference is represented below each blot. (B) Cell growth of U138-GGT7 with reduced expression of GGT7 under low nutrient growth conditions. [file 12885_2015_1232_MOESM2_ESM.pptx]
